# Supplementary material for: Adding a Leafy Vegetable Fraction to Diets Decreases the Risk of Red Meat Mortality in MASLD Subjects: Results from the MICOL Cohort
Source: Nutrients. 2024 Apr 18;16(8):1207. doi: 10.3390/nu16081207 (PMC11053907; doi:10.3390/nu16081207)
Supplement: Supplementary file 1 [file nutrients-16-01207-s001.zip › Supplementary Table S1.pdf]

Table S1. Single food composition of food groups used.

Food Group	Sample foods
(1) Red Meat	Veal; Horse; Pork; Liver; Lamb
(2) Leafy Vegetables	Spinach; Chard; Chicory; Salad
