# Supplementary material for: Adding a Leafy Vegetable Fraction to Diets Decreases the Risk of Red Meat Mortality in MASLD Subjects: Results from the MICOL Cohort
Source: Nutrients. 2024 Apr 18;16(8):1207. doi: 10.3390/nu16081207 (PMC11053907; doi:10.3390/nu16081207)
Supplement: Supplementary file 1 [file nutrients-16-01207-s001.zip › Supplementary Table S2.pdf]

Table S2. Malignant tumors (ICD-10) (*n=171*).

Tumors *	%
Lip, Oral Cavity, Pharynx, Skin and Soft Tissue	12 (7.02)
Digestive System	66 (38.60)
Respiratory System and Intrathoracic Organs	29 (16.96)
Breast	9 (5.26)
Female and Male Genital Organs, and Urinary system	28 (16.37)
Lymphatic Tissue, Hematopoietic Tissue and Related Tissues	17 (9.94)
Others Tumors	10 (5.85)

* As frequency and percentage (%).
