# Supplementary figures and images for: Adding a Leafy Vegetable Fraction to Diets Decreases the Risk of Red Meat Mortality in MASLD Subjects: Results from the MICOL Cohort
Source: Nutrients. 2024 Apr 18;16(8):1207. doi: 10.3390/nu16081207 (PMC11053907; doi:10.3390/nu16081207)

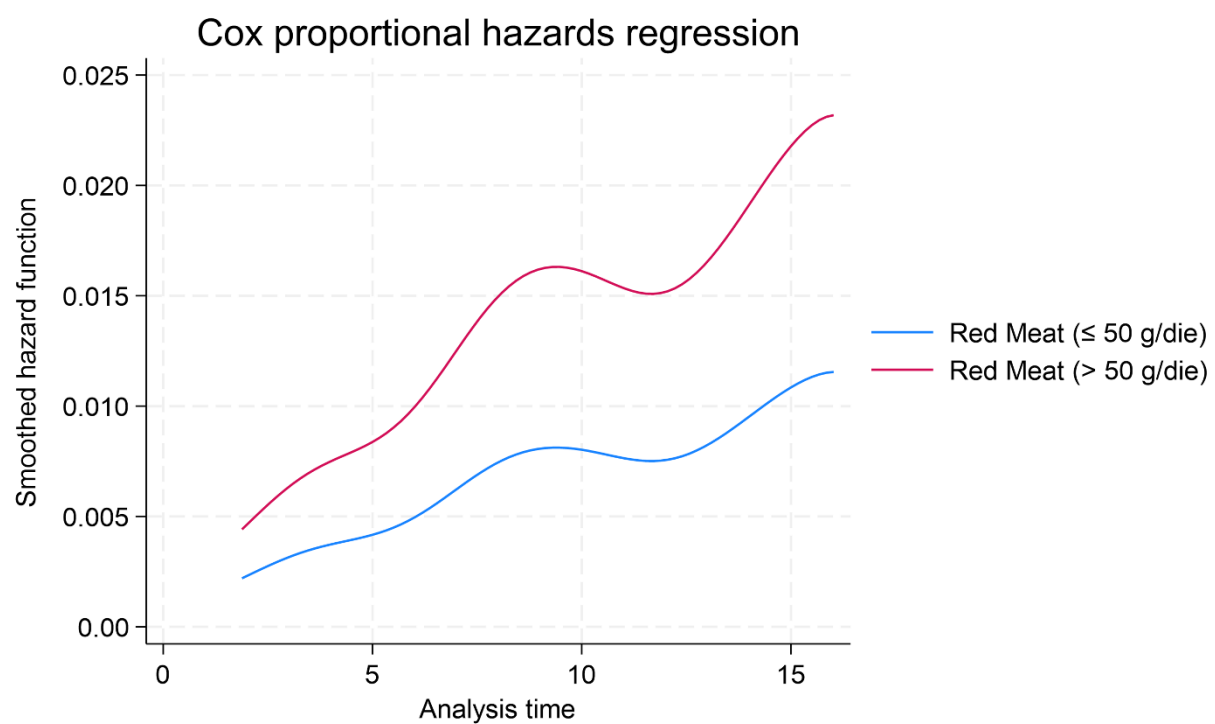

**Figure S1.** Cumulative hazard function stratified by red meat consumption.

Supplement: Supplementary file 1 [file nutrients-16-01207-s001.zip › Supplementary Figure S1.pdf]
